# Assembly and characterization of the complete mitochondrial genome of *Setaria viridis* (L.) Beauv

**DOI:** 10.3389/fpls.2026.1882896

**Published:** 2026-06-22

**Authors:** Xin Ning, Zhiyuan Li, Bo Wang, Qing Chen, Huiling Wu, Jiewei Zhang

**Affiliations:** 1 Beijing Key Laboratory of Agricultural Genetic Resources and Biotechnology, Beijing Key Laboratory of Crop Molecular Design and Intelligent Breeding, Beijing Academy of Agriculture and Forestry Sciences, Beijing, China; 2 Key Laboratory of Photobiology, Institute of Botany, Photosynthesis Research Center, Chinese Academy of Sciences, Beijing, China; 3 School of Life Sciences, Shandong Yantai University, Yantai, China

**Keywords:** chloroplast-derived fragments, mitochondrial genome, phylogenetic analysis, repeat sequences, RNA editing, *Setaria viridis*

## Abstract

*Setaria viridis* (L.) Beauv. is rapidly emerging as a model plant within the *Panicoideae* subfamily of *Poaceae* for studying C4 photosynthesis. The ‘Fangshan’ ecotype of *S. viridis* exhibits high adaptability to drought and nutrient-poor environments and holds historical significance in traditional Chinese medicine. Nevertheless, cytoplasmic genomic resources for this species, particularly its mitochondrial genome, remain entirely uncharacterized. To address this gap, we assembled the complete mitochondrial genome of the ‘Fangshan’ ecotype *de novo* using a hybrid approach combining Oxford Nanopore long reads and Illumina short reads. The mitochondrial genome adopts a predominantly single circular conformation with a total length of 437, 906 bp and a GC content of 43.96%, containing 33 unique protein-coding genes, 20 tRNA genes, and 3 rRNA genes. Furthermore, 111 Simple Sequence Repeats (SSRs), 33 tandem repeats, and 493 pairs of dispersed repeats were identified. Analysis revealed 31 plastid-derived DNA sequences integrated into the mitochondrial genome, constituting 3.19% of its total length. These sequences include one protein-coding gene and nine tRNA genes. Phylogenetic reconstruction demonstrated that the *S. viridis* clusters closely with *S. italica*, and Poaceae species form a distinct clade. Subsequently, in *silico* prediction identified 463 potential RNA editing sites (exclusively C-to-U conversions) across the 33 mitochondrial protein-coding genes. *ccmC*, *ccmFN*, and *nad2* carried the highest numbers of predicted editing sites (36, 30, and 30, respectively). As core components of the mitochondrial electron transport chain, extensive editing in these genes may reflect strong functional constraints essential for energy metabolism during foxtail millet domestication. This study establishes a foundational genomic resource for investigating cytoplasmic genome evolution and the genetic basis of environmental adaptation in the genus *Setaria*.

## 1 Introduction

The Poaceae family provides over 70% of human caloric intake, with *Oryza sativa*, *Triticum aestivum*, and *Zea mays* predominating, along with minor cereals including *Setaria italica*, *Sorghum bicolor*, *Avena sativa*, *Hordeum vulgare*, *Secale cereale*, and *Panicum miliaceum* (Franco et al., 2025; Ji et al., 2024). *Setaria viridis* (L.) Beauv., Green foxtail, also called green millet, belongs to *Setaria* Beauv. It is the ancestor and domesticated relative of cultivated foxtail millet, which is of great importance to dry land agriculture and has been grown in China for >10,500 years (Jia et al., 2013; Yang et al., 2012). The *S. viridis*, with its compact genome of approximately 500 Mb, diploid, and short life cycle of 8-10 weeks, exhibits high self-compatibility and seed yield (Li and Brutnell, 2011). Its amenability to efficient transformation, sensitivity to CRISPR-Cas9-mediated mutagenesis, and ease of laboratory cultivation is driving its emergence as a key model system for studying panicoid grasses and C4 photosynthesis (Mamidi et al., 2020; Martins et al., 2015). The genetic and genomic elucidation of the *S. viridis* will help improve breeding programs in those close relative crops. While current research predominantly focuses on the nuclear and chloroplast genome or functional analysis of specific genes in the *S. viridis*, the structure and sequence of its mitochondrial genome remain uncharacterized, impeding comprehensive studies on cytoplasmic inheritance in this key model species (Franco et al., 2025; Jia et al., 2013; Mamidi et al., 2020; Thielen et al., 2020; Wang and Gao, 2016; Yang et al., 2021; Yu et al., 2023; Zhang et al., 2012). Here, this study aims to comprehensively analyze the structural and sequence features of the *S. viridis* mitogenome. This work will enrich mitochondrial genomic resources for the *S. viridis*, thereby providing essential groundwork for understanding its molecular regulatory networks and advancing genetic breeding in closely related species.

Plant mitogenomes exhibit extraordinary structural dynamism, characterized by large sizes (>200 kb), multipartite and plastic architectures, large repetitive sequences and extensive genomic rearrangements (Guo et al., 2023; Schuster and Brennicke, 1994; Wynn and Christensen, 2019; Xia et al., 2023; Zhou et al., 2023). Frequent recombination mediated by long repetitive sequences (>1 kb) generates alternative conformations (Ke et al., 2023; Lu and Li, 2023; You et al., 2022). Horizontal gene transfers (HGTs) from plastids (MTPTs) and nuclei (NUMTs), contribute to gene content variation (Bock, 2010; Yang et al., 2023; Zhao et al., 2024). Furthermore, pervasive RNA editing (C-to-U conversions) modulates transcript functionality, which further contributes to functional diversification (Guo et al., 2023; Liu et al., 2022b).

However, while mitogenomes of crops like rice and related grasses like *Poa pratensis*, *Avena longiglumis* and foxtail millet have been sequenced (Ai et al., 2023; Liu et al., 2023a; Notsu et al., 2002; Yang et al., 2020; Zhang et al., 2024), the evolutionary trajectory of mitochondrial genomes within the *Setaria* lineage remains unexplored. The absence of a high-quality *S. viridis* assembly precludes comparative analyses of structural evolution, RNA editing landscapes, and lineage-specific innovations within the Panicoideae subfamily. Technical limitations further exacerbate this gap. Short-read sequencing often fails to resolve long repeats, leading to fragmented assemblies that obscure recombination-derived isoforms (Fischer et al., 2022). The *S. viridis* mitogenome-with its uncharacterized repetitive landscape and putative multichromosomal structure-demands long-read technologies for accurate reconstruction.

In this study, we assembled the first complete mitochondrial genome of *S. viridis* using Oxford Nanopore and Illumina reads. The ‘Fangshan’ ecotype, collected from a semi-arid region in Beijing, exhibits dwarf stature, profuse tillering, and strong drought and nutrient tolerance, making it ideal for C4 research. This accession was selected due to its local adaptation, available nuclear and transcriptomic data, and typical C4 traits, allowing investigation of mitochondrial roles in energy metabolism and adaptation. We characterized GC content, codon usage bias, repeats, sequence migration, and predicted C-to-U RNA editing sites. Phylogenetic relationships among Panicoid grasses were resolved using conserved mitochondrial protein-coding genes. The assembled mitogenome serves as a foundation for functional studies and provides a genomic resource for harnessing cytoplasmic diversity in Setaria improvement and understanding mitogenome evolution in C4 plant.

## 2 Materials and methods

### 2.1 Plant materials, DNA extraction and sequence

The inbred line ‘Fangshan’ ecotype of *S. viridis*, utilized in this study, is deposited within the Beijing Crop Germplasm Resources Infrastructure, located in Haidian Distrct, Beijing, China (39°35′N, 116°05′E). Fully matured fresh leaves of the *S. viridis* plants were collected from a greenhouse at BAAFS after 1 month of growth. Subsequently, the leaves were meticulously cleaned with DEPC-treated water and preserved at a temperature of -80°C in a refrigerator. Genomic DNA was extracted using the FastPure^®^ Plant DNA Isolation Mini Kit (DC104, Vanzyme Biotech, Nanjing, China). The DNA sample was subsequently sent to a sequencing company, where a hybrid sequencing strategy combining Illumina short-read and Oxford Nanopore Technologies (ONT) long-read platforms was adopted. The resulting paired-end Illumina reads and ONT long reads were generated and used for downstream hybrid assembly.

### 2.2 Assembly and annotation methods of plant mitochondrial genome

Using long-read data, the mitochondrial genome was assembled. The Flye software, configured with default parameters, was employed to directly assemble the long-read sequencing data, resulting in a graphical assembly in GFA format (Kolmogorov et al., 2019). For all contigs in fasta format from the assembly, we used “makeblastdb” to build a database. Subsequently, the BLASTn program was employed, with the mitochondrial genes of closely related species (*Setaria italica*, OR734218.1-OR734219.1; *Zea mays*, NC_007982.1; *Tripsacum dactyloides*, NC_008362.1; *Paspalum vaginatum*, NC_086594.1; *Saccharum narenga*, NC_088484.1) as query sequences to identify contig fragments containing the mitochondrial genome. The parameters were “-evalue 1e-5 -outfmt 6 -max_hsps 10 -word_size 7 -task blastn-short”. The Bandage software (v0.8.1) was utilized to visualize the GFA file. Mitochondrial contigs were subsequently filtered based on BLASTn results, leading to the creation of a draft map of the mitochondrial genome (Wick et al., 2015). Minimap2 software (v2.26-r1175) was employed to map both long-read and short-read data to the mitochondrial contigs (Li, 2018). Reads mapped to the mitochondria were filtered out and saved separately for subsequent hybrid assembly. By integrating both short-reads and long-reads sequencing data, a hybrid assembly strategy was employed to construct the mitochondrial genome. Using Unicycler with default parameters, we achieved a hybrid assembly and obtained the mitochondrial genome (Wick et al., 2017). Subsequently, the Bandage software was employed to visualize the mitochondrial genome.

For the annotation of protein-coding genes in the mitochondrial genome, mitochondrial genomes of closely related species were selected as reference genomes. The plant mitochondrial genome annotation tool PMGA (http://www.1kmpg.cn/pmga/) was used to annotate the mitochondrial genome (Li et al., 2025). PMGA demonstrates a relatively effective performance in annotating genes at splice sites and during trans-splicing. For tRNA within the mitochondrial genome, the tRNAscan-SE software (v2.0.11) was employed for annotation (Lowe and Eddy, 1997). For rRNA in the mitochondrial genome, the BLASTn software (v2.13.0) was utilized for annotation (Chen et al., 2015). Any annotation errors in the mitochondrial genome were manually corrected using the Apollo software (v1.11.8) (Lewis et al., 2002). The OGDRAW software was used to visualize the mitochondrial genome map (Greiner et al., 2019).

### 2.3 Codon bias

The CDSs were extracted from the annotated mitochondrial genome using PhyloSuite (v1.1.16) (Zhang et al., 2020). Subsequent codon bias analysis was performed in MEGA v7.0 (Kumar et al., 2016). The RSCU was calculated. RSCU >1 indicates positive bias, RSCU <1 negative bias, and RSCU = 1 neutral usage. This approach delineates codon preference patterns relevant to translational efficiency and evolutionary adaptation.

### 2.4 Repeat sequence and mitochondrial genome structure

Repeat sequences within the mitochondrial genome of the *S. viridis* was characterized using a multi-tool approach. Microsatellites (SSRs) were identified using MISA (v2.1) with default parameters (Beier et al., 2017). Tandem repeats were detected with TRF (v4.09) using standard parameters optimized for plant mitochondrial genomes (Benson, 1999). Dispersed repeats were identified via the REPuter web server with appropriate similarity thresholds and window sizes to minimize false positives (Kurtz et al., 2001). Outputs from these tools were collated and quantified using Microsoft Excel to determine abundances and length distributions. The processed data were then visualized using Circos (v0.69.9), generating circular genome plots where distinct colors represented the spatial distribution and abundance of each repeat type (Zhang et al., 2013).

To resolve repetitive regions in the graph-based assembled mitochondrial genome, long-read sequencing data were leveraged. Long reads were mapped onto identified repeats to detect reads spanning these regions. For each repetitive locus, alignments were examined for spanning reads. Using these spanning reads, the most plausible traversal paths through the repeats were inferred, enabling reconstruction of the mitochondrial genome’s structure. This approach utilized the long-range continuity of long reads to overcome ambiguity caused by repeats, providing accurate structural resolution essential for functional and evolutionary analyses.

### 2.5 Sequence migration

Sequence migration analysis between mitochondrial and chloroplast genomes requires high-quality chloroplast genome data. The chloroplast genome was assembled *de novo* using GetOrganelle and annotated with CPGAVAS2 (Jin et al., 2020; Shi et al., 2019). Manual annotation refinement was performed using CPGView to ensure accuracy (Liu et al., 2023b). Potential sequence migration events were identified by pairwise BLASTn (v2.13.0) alignments between the organellar genomes, applying an e-value threshold of 1e-5 (Chen et al., 2015). Significant homologous regions were visualized as connecting links between the mitochondrial and chloroplast genomes using Circos (v0.69.9) to identify migration patterns (Zhang et al., 2013).

### 2.6 Methodology for phylogenetic

Based on genetic affinities, mitochondrial genomes of closely related species were selected and retrieved. Shared protein-coding genes were extracted from these genomes using PhyloSuite (v1.1.16) to establish a conserved gene set for phylogenetic analysis (Zhang et al., 2020). Multiple sequence alignment was performed with MAFFT (v7.505) to ensure accurate homology positioning (Katoh and Standley, 2013). Phylogenetic reconstruction was conducted under the maximum likelihood framework using IQ-TREE (v1.6.12), with branch support assessed by 1,000 SH-like approximate likelihood ratio tests (-alrt 1000) and 1,000 ultrafast bootstrap replicates (-B 1000) (Nguyen et al., 2015). Final trees were visualized and annotated using iTOL (v6) for evolutionary interpretation (Letunic and Bork, 2019).

### 2.7 RNA editing events

To identify RNA editing events, the DNA sequences of all PCGs encoded by the mitochondrial genome of the target species were first compiled as input files. Then, the Deepredmt tool, which is underpinned by a Convolutional Neural Network (CNN)-based predictive model, was deployed to predict C-to-U RNA editing sites within these mitochondrial PCGs (Edera et al., 2021). Compared with traditional RNA editing prediction tools, Deepred-mt exhibits superior accuracy in site prediction due to its advanced deep-learning architecture. In order to ensure the reliability of predicted editing events, only those results with a predicted probability value greater than 0.9 were retained for subsequent analysis.

### 2.8 Synteny analysis

To conduct synteny analysis, the BLASTn program was employed to identify conserved homologous sequences, referred to as syntenic blocks. The parameter settings for BLASTn were configured as follows: an E-value threshold of 1e-5 was applied to filter statistically insignificant matches, a word size of 9 was specified to initiate sequence alignment, gap opening and extension penalties were set to 5 and 2, respectively, while match and mismatch rewards/penalties were defined as 2 and -3. To ensure the reliability and biological relevance of subsequent analyses, only syntenic blocks with a length exceeding 500 bp were retained for further investigation. Subsequently, the MCscanX software, a specialized tool for detecting genomic collinearity, was utilized (Wang et al., 2012). Leveraging the output results generated from the BLASTn-based homology searches, MCscanX was used to perform pairwise comparisons and construct Multiple Synteny Plots. Through this workflow, conserved syntenic blocks across genomes were visualized, enabling the systematic exploration of genomic structural relationships and evolutionary rearrangements at the sequence level.

## 3 Results

### 3.1 Sequence assembly and annotation

**Figure 1:**
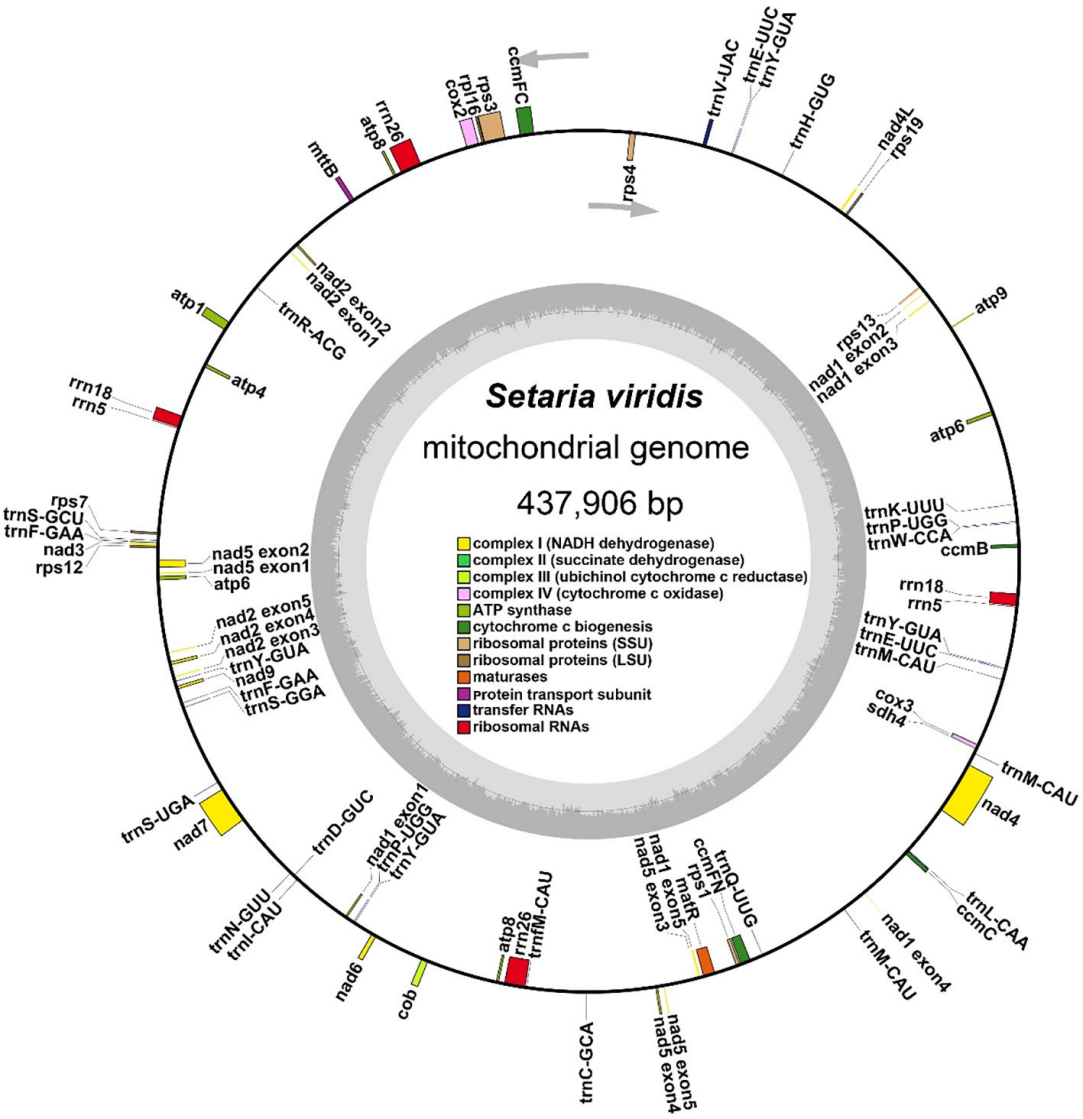
Mitochondrial genome map of the *S. viridis*. Linearized map of the circular mitochondrial genome of the *S. viridis*. A horizontal black line indicates the linearized axis of the genome. The colored rectangles, lines, and symbols above the axis represent the positions, lengths, and transcriptional directions of annotated features. Colors correspond to functional categories shown in the legend on the right. The color key defines each functional gene category as follows: complex I (blue), complex II (yellow), complex III (orange), complex IV (purple), cytochrome c biogenesis (light blue), ribosomal proteins SSU (green), ribosomal proteins LSU (dark green), maturases (pink), protein transport subunit (brown), tRNAs (red), and rRNAs (dark blue). The outer and inner circles represent the forward and reverse strand transcription directions, respectively. Additional features, including repeats and the GC content curve, are also labeled on the map.

To analysis the structure of the *S. viridis* mitochondrial genome, *de novo* assembly was progressed using Flye based on the long-reads raw data. The resulting contigs were selected with core mitochondrial genes using BLASTn. The assembly results revealed that the mitochondrial genome of the *S. viridis* primarily exists as a single circular molecule, with a total length of 437, 906 bp and a GC content of 43.96% (**Figure 1**). After annotation on this mitochondrial genome, a total of 33 unique protein-coding genes were identified, among which 2 genes are present in multiple copies. These protein-coding genes include 24 distinct mitochondrial core genes and 9 non-core genes. A total of 20 tRNA genes were annotated, including five that are multi-copy. Additionally, three rRNA genes were identified, all of which are also multi-copy (**Supplementary Table 1**). The core genes comprise a diverse array of functional genes essential for mitochondrial processes. The ATP synthase genes, specifically *atp1*, *atp4*, *atp6*, *atp8*, and *atp9*, are crucial for ATP production, the cellular process responsible for generating energy. The nine NADH dehydrogenase genes (*nad1*, *nad2*, *nad3*, *nad4*, *nad4L*, *nad5*, *nad6*, *nad7*, and *nad9*) are essential components of the mitochondrial electron transport chain, directly participating in NADH oxidation and playing a pivotal role in cellular energy metabolism. The four cytochrome C biogenesis genes-*ccmB*, *ccmC*, *ccmFC*, and *ccmFN-*are crucial for the proper synthesis and function of cytochrome C, a vital component of the respiratory chain. The three cytochrome C oxidase genes-*cox1*, *cox2*, and *cox3*-are essential for the terminal step of the electron transport chain, facilitating oxygen reduction. Additionally, the *mttB* gene encodes a membrane transport protein that facilitates molecule transport across the mitochondrial membrane. The *matR* gene, a maturase, assists in the maturation of mitochondrial RNAs, while the *cob* gene, encoding ubiquinol-cytochrome C reductase, is integral to the respiratory chain complex. The non-core genes also play significant roles within the mitochondrial system. One ribosomal large subunit gene, *rpl16*, and seven ribosomal small subunit genes-*rps1*, *rps3*, *rps4*, *rps7*, *rps12*, *rps13*, and *rps19*-are essential for assembling and functioning mitochondrial ribosomes, which are vital for protein synthesis in mitochondria. Furthermore, one succinate dehydrogenase gene, *sdh4*, contributes to the citric acid cycle and electron transport, supporting the plant’s overall energy metabolism.

### 3.2 Codon bias

**Figure 2:**
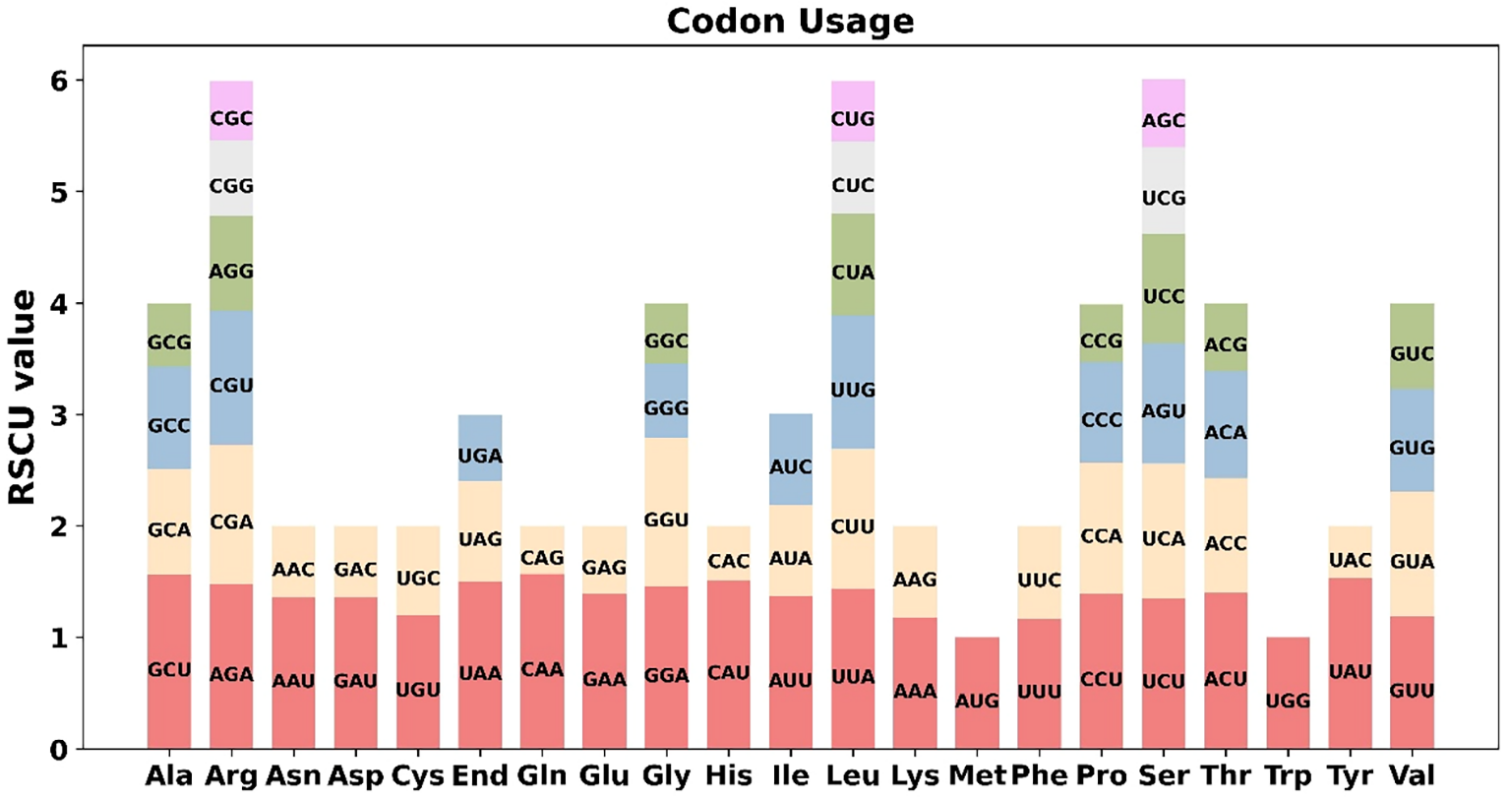
The *S. viridis* mitogenome RSCU. The X-axis represents codon families, while RSCU values indicate the frequency of a specific codon relative to the expected frequency under uniform synonymous codon usage.

A codon bias analysis was conducted on the 33 unique PCGs within the mitochondria of the *S. viridis*. The utilization of codons for each amino acid is detailed in **Table 1**. Codons with a RSCU value exceeding 1 are deemed to be preferentially utilized by their respective amino acids. As illustrated in **Figure 2**, apart from the start codon AUG and the codon UGG for tryptophan (Trp), both having an RSCU value of 1, there is a notable codon usage bias in the mitochondrial PCGs. For example, glutamine (Gln) exhibits a significant preference for the codon CAA, with its RSCU value being the highest among all codons in these mitochondrial PCGs, reaching 1.57.

**Table 1:** The relative synonymous codon usage of each amino acid in the *S. viridis* mitochondrial genome.

Amino	Codon 1	Codon 2	Codon 3	Codon 4	Codon 5	Codon 6
	RSCU	RSCU	RSCU	RSCU	RSCU	RSCU
Ala	GCU	GCA	GCC	GCG		
	1.56	0.95	0.92	0.57		
Arg	AGA	CGA	CGU	AGG	CGG	CGC
	1.48	1.25	1.2	0.85	0.68	0.53
Asn	AAU	AAC				
	1.36	0.64				
Asp	GAU	GAC				
	1.36	0.64				
Cys	UGU	UGC				
	1.2	0.8				
End	UAA	UAG	UGA			
	1.5	0.9	0.6			
Gln	CAA	CAG				
	1.57	0.43				
Glu	GAA	GAG				
	1.39	0.61				
Gly	GGA	GGU	GGG	GGC		
	1.46	1.33	0.67	0.54		
His	CAU	CAC				
	1.51	0.49				
Ile	AUU	AUA	AUC			
	1.37	0.82	0.82			
Leu	UUA	CUU	UUG	CUA	CUC	CUG
	1.44	1.25	1.2	0.91	0.65	0.54
Lys	AAA	AAG				
	1.18	0.82				
Met	AUG					
	1					
Phe	UUU	UUC				
	1.17	0.83				
Pro	CCU	CCA	CCC	CCG		
	1.39	1.18	0.9	0.52		
Ser	UCU	UCA	AGU	UCC	UCG	AGC
	1.35	1.21	1.08	0.98	0.78	0.61
Thr	ACU	ACC	ACA	ACG		
	1.4	1.03	0.96	0.61		
Trp	UGG					
	1					
Tyr	UAU	UAC				
	1.53	0.47				
Val	GUU	GUA	GUG	GUC		
	1.19	1.12	0.92	0.77		

The observed codon usage bias suggests that during mitochondrial gene expression and protein synthesis in the *S. viridis*, there are specific preferences for certain codons to encode amino acids. These preferences may be influenced by various factors. On one hand, they may be linked to translation efficiency. Codons with higher RSCU values could be recognized and translated more effectively by mitochondrial ribosomes, facilitating the rapid synthesis of mitochondrial proteins and maintaining normal mitochondrial functions. On the other hand, from an evolutionary standpoint, this codon bias may be attributed to the long-term adaptive evolution of the mitochondrial genome, which aids the plant in better adapting to its specific ecological environment and physiological requirements. Further in-depth studies, including the analysis of mitochondrial ribosome binding characteristics and translation kinetics, can enhance our understanding of the biological significance underlying this codon usage bias.

### 3.3 Repeat sequence

**Figure 3:**
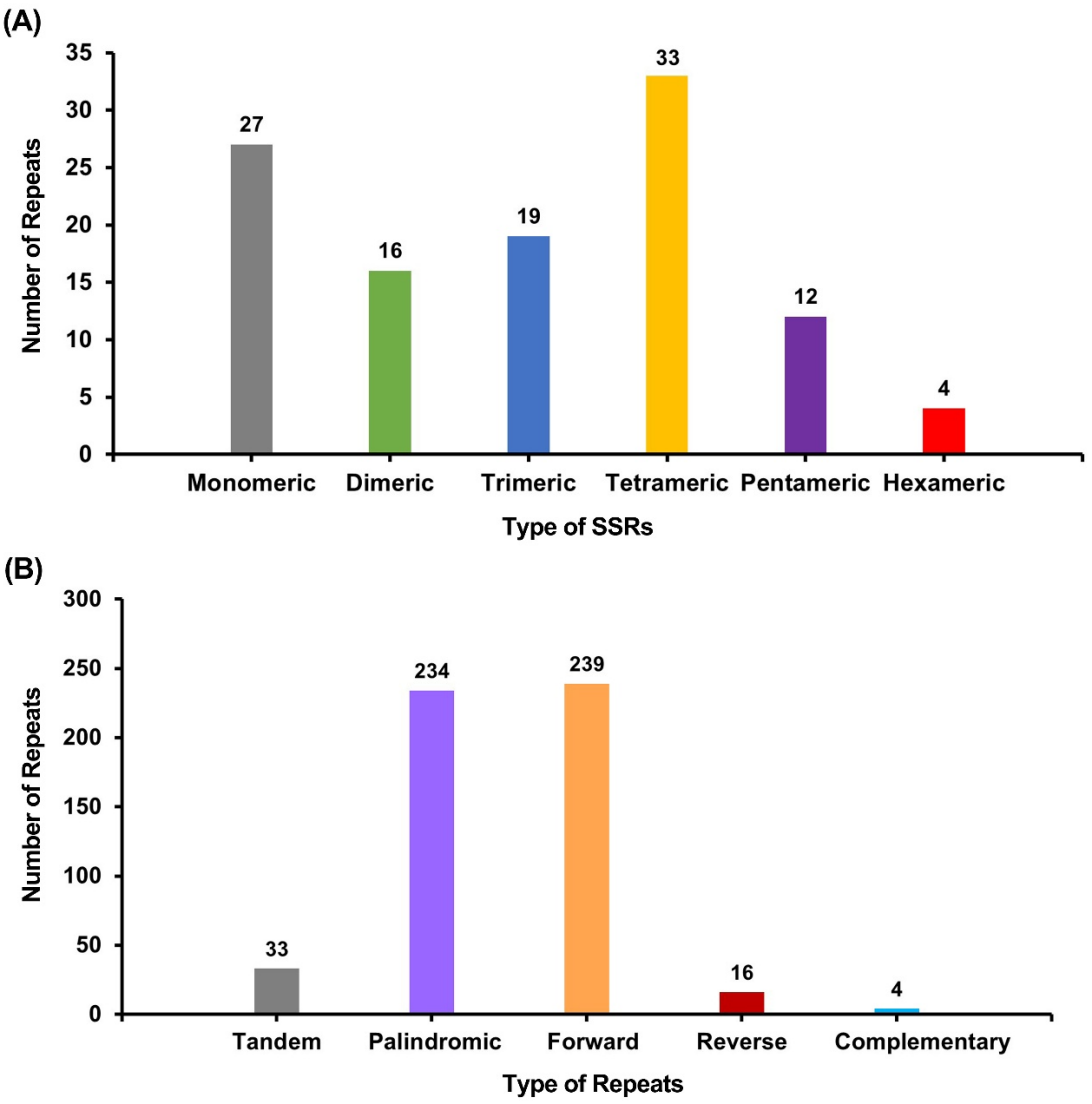
Detected repeats in the *S. viridis*. **(A)** The X-axis denotes mitochondrial molecules, while the Y-axis indicates the frequency of repetitive fragments. Monomeric SSRs are denoted by gray, dimeric SSRs by green, trimeric SSRs by blue, tetrameric SSRs by yellow, pentameric SSRs by purple, and hexameric SSRs by red. **(B)** the X-axis represents mitochondrial molecules, and the Y-axis represents the occurrence of repetitive fragments. Tandem repeats are shown in gray, palindromic repeats in purple, forward repeats in orange, reverse repeats in red, and complementary repeats in blue.

This study systematically characterized repeat sequences in the mitochondrial genome of the *S. viridis*, including SSRs, tandem repeats, and dispersed repeats (**Figure 3**). A total of 111 SSRs were identified (**Supplementary Table 2**). Among them, mononucleotide and dinucleotide repeats constituted 38.74% of the total SSRs, highlighting the significant presence of short-length repeat motifs within the genome’s repetitive elements. Notably, T-rich mononucleotide repeats were particularly abundant: out of the 27 mononucleotide SSRs detected, 13 (48.15%) were T-repeat units. This prevalence of T-mononucleotide SSRs may indicate strand-specific compositional biases in the mitochondrial genome, which could influence local DNA structure and replication dynamics. In the mitochondrial genome of the *S. viridis*, 33 tandem repeats (satellite DNA) were identified, each surpassing a 75% matching degree and varying in length from 5 to 102 base pairs (**Supplementary Table 3**). These repeats may function as ‘hotspots’ for genomic rearrangements, as tandem repeat-mediated slipped-strand mispairing or unequal crossing-over can induce structural variations during mitochondrial evolution. Dispersed repeats were identified via sequence similarity-based screening, yielding a total of 493 repeat pairs with lengths ≥30 bp, which were categorized into four structural types (**Supplementary Table 4**). These include palindromic repeats (234 pairs, longest 8, 135 bp), forward repeats (239 pairs, longest 816 bp), reverse repeats (16 pairs), and the rarest complementary repeats (4 pairs).

The diversity and abundance of repeat sequences in the mitochondrial genome of the *S. viridis* indicate that repeat-mediated mechanisms significantly influence mitochondrial genome structure. These mechanisms affect various functions, including replication fidelity, gene expression regulation, and evolutionary divergence. This research lays the groundwork for future studies examining the functional implications of repeat-associated genomic plasticity in grass mitochondrial genomes.

### 3.4 Mitochondrial genome structure

**Figure 4:**
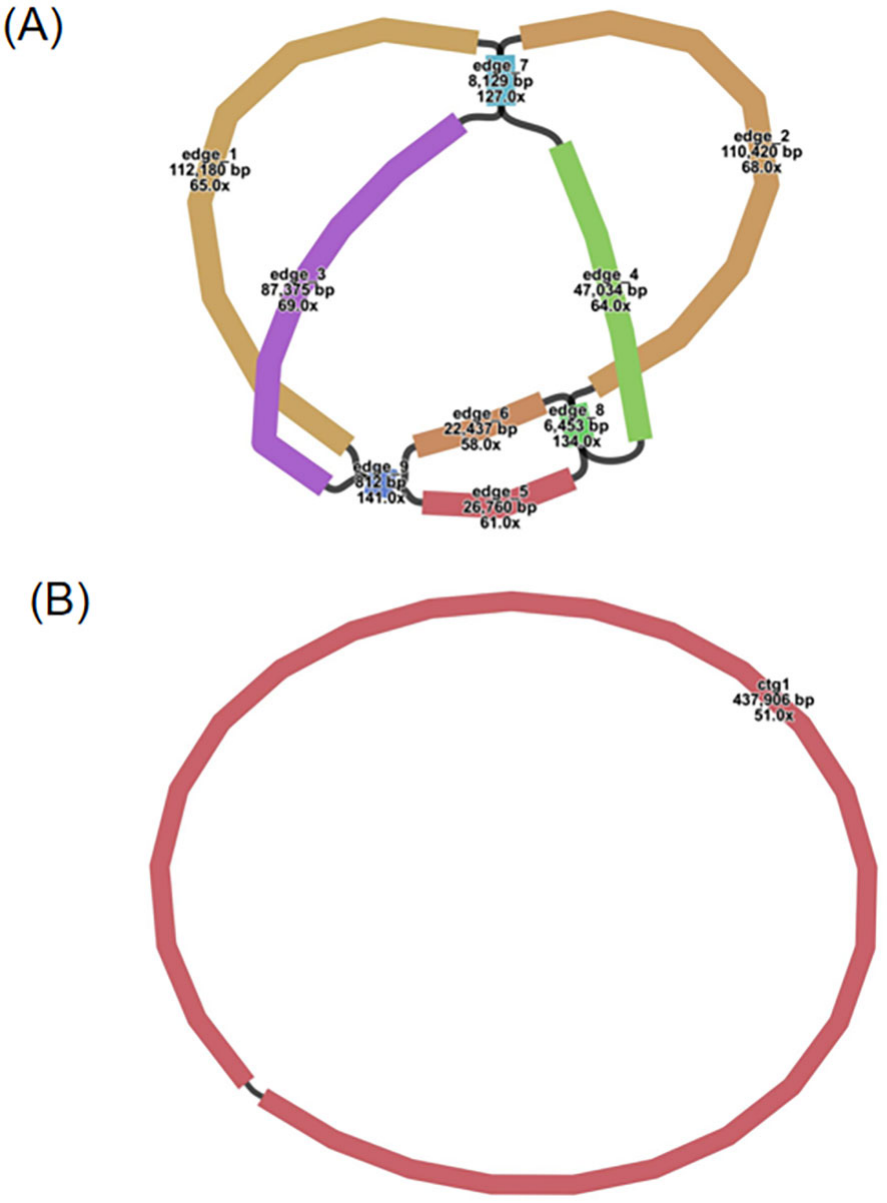
The assembly graph of the *S. viridis* mitogenome. **(A)** Schematic diagram of mitochondrial genome assembly based on ONT data assembled with Flye (with node IDs labeled in the figure). **(B)**Schematic representation of the hybrid assembly strategy for the mitochondrial genome using Unicycler, integrating both ONT and Illumina data (node IDs are labeled in the figure).

To characterize the mitochondrial genome of the *S. viridis*, we employed integrated long-read assembly and visualization strategies. Initial long-read (ONT) assembly with Flye generated 9 contigs (**Supplementary Table 5**), whose topological relationships and repeat-associated complexities were visualized *via* Bandage (**Figure 4A**), revealing repetitive regions that hinder linear assembly. To resolve these complexities, a hybrid assembly was performed using Unicycler, which integrates error-filtered ONT long reads (for continuity) and Illumina short reads (for base accuracy). This approach produced a complete circular mitochondrial genome (**Figure 4B**) by traversing repetitive regions *via* a defined path, resolving ambiguities introduced by repeats. In contrast to the error-prone Flye assembly (relying solely on uncorrected ONT data), the Unicycler assembly achieved high base accuracy, suitable for downstream analysis. The circular structure, a canonical feature of plant mitochondrial genomes, validated assembly completeness. Repeat regions resolved here likely represent evolutionary hotspots driving genomic rearrangements. Overall, our workflow-combining Flye scaffolding, Bandage visualization, and Unicycler error correction-effectively reconstructed the *S. viridis* mitochondrial genome, providing a robust foundation for subsequent gene content, RNA editing, and evolutionary analyses.

### 3.5 Mitochondrial plastid DNAs

**Figure 5:**
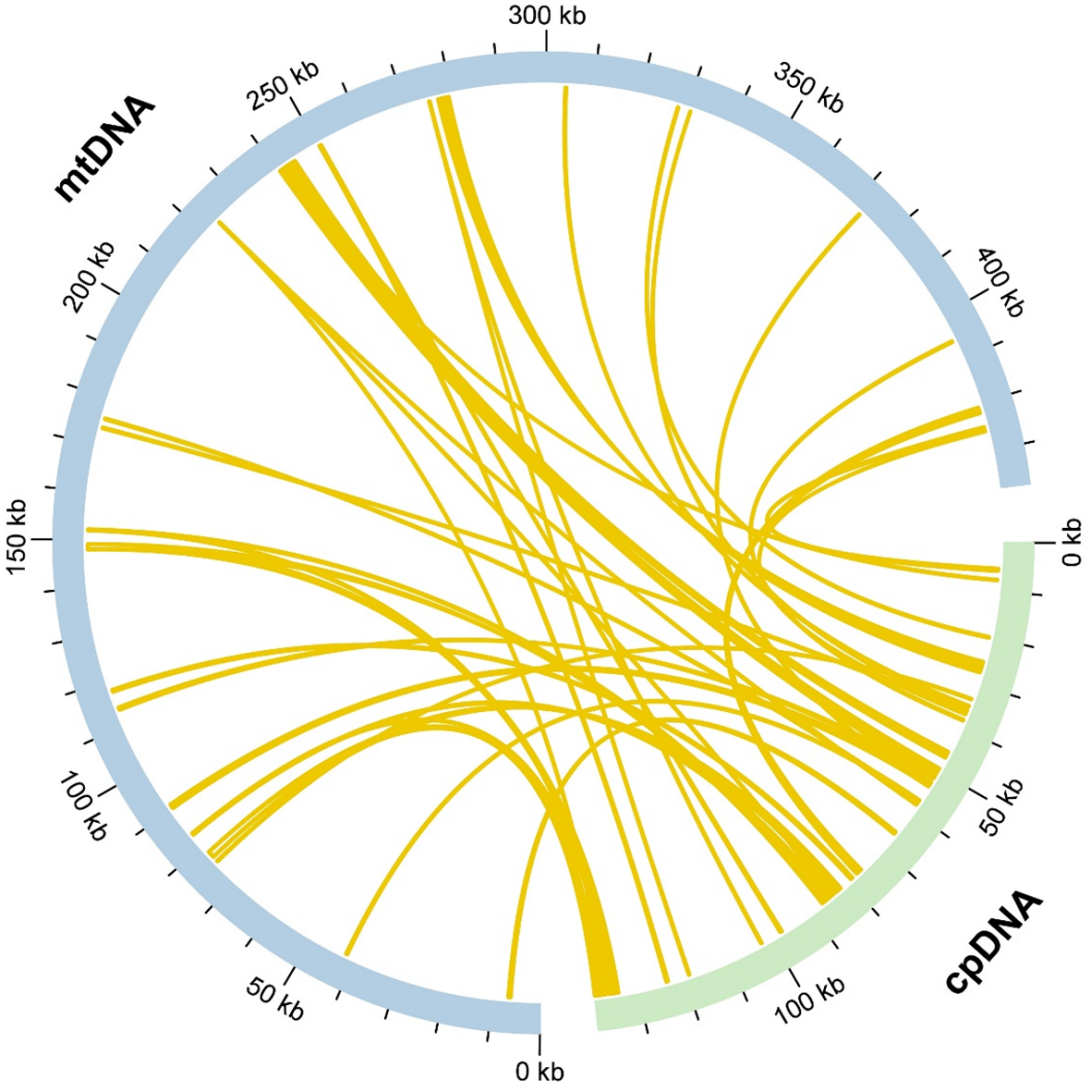
Schematic diagram of gene transfer between chloroplast and mitochondrial genomes in the *S. viridis*. The blue arc represents the mitochondrial genome, the green arc represents the chloroplast genome, and the yellow connecting lines between the arcs correspond to homologous genomic fragments.

Comprehensive homology analysis identified 31 distinct regions of sequence homology between the mitochondrial (mtDNA) and chloroplast (cpDNA) genomes in the *S. viridis* (**Figure 5**). These horizontally transferred sequences collectively span 13, 972 bp, representing 3.19% of the mtDNA length. Fragment sizes varied considerably, with the largest transfer (MTPT1) encompassing 2, 292 bp, indicative of substantive inter-organellar DNA migration consistent with patterns observed across angiosperms. Functional annotation revealed these mtDNA-integrated sequences harbor 10 intact organellar genes originating from the chloroplast: the protein-coding gene *ndhJ* (encoding a subunit of plastid NADH dehydrogenase) and nine tRNA genes (*trnC-GCA*, *trnF-GAA*, *trnH-GUG*, *trnM-CAU*, *trnN-GUU*, *trnP-UGG*, *trnS-GGA*, *trnV-UAC*, *trnW-CCA*) (**Supplementary Table 6**). The acquisition of plastid tRNA genes by the mitochondrion suggests a potential mechanism for supplementing the mt tRNA repertoire.

### 3.6 Phylogenetic analysis and synteny analysis

**Figure 6:**
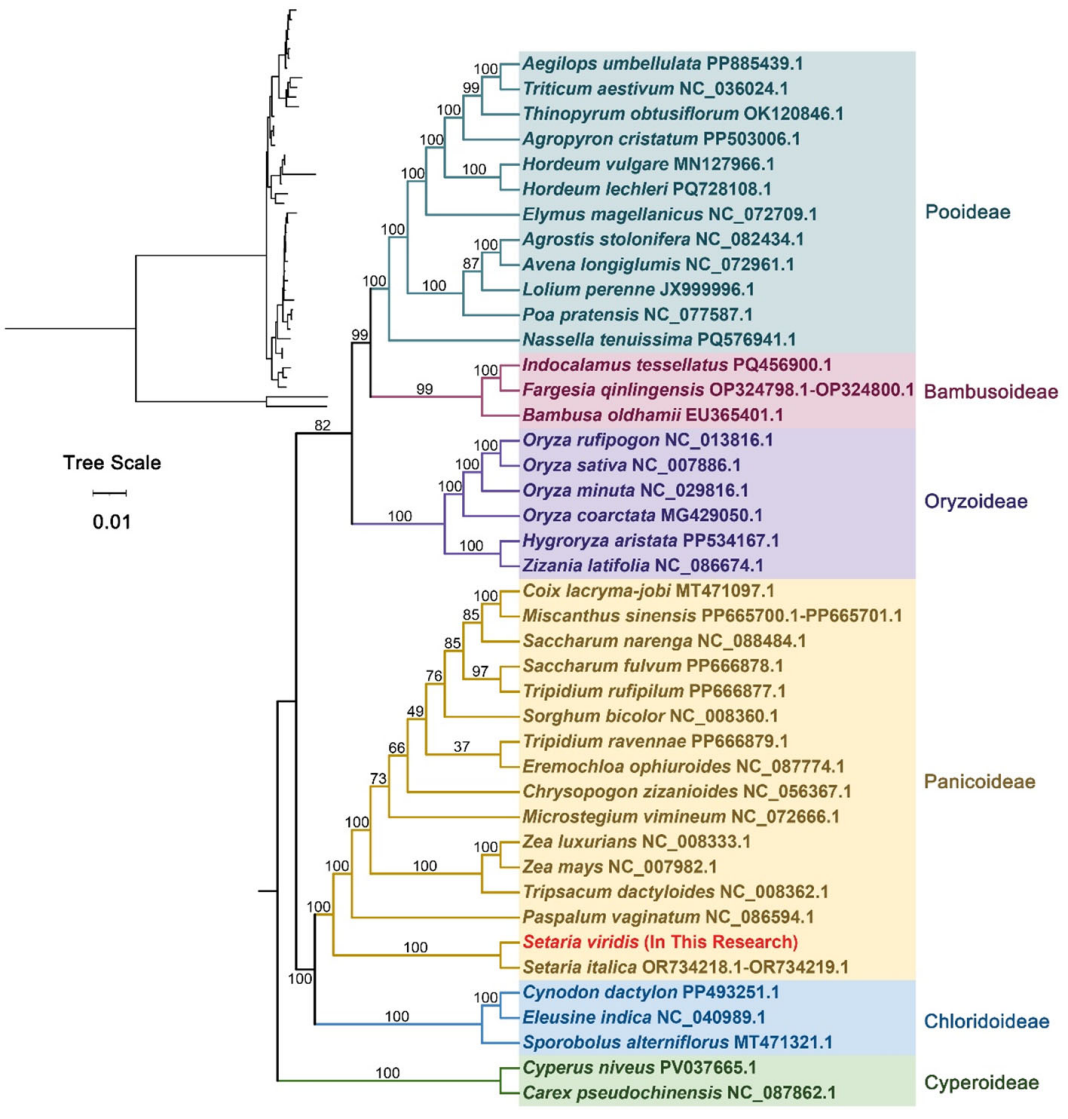
Phylogenetic analysis of the *S. viridis* mitochondrial genome. Bootstrap support values are shown on each node, with colors representing the respective plant families.

A phylogenetic tree was reconstructed for 42 species (**Supplementary Table 7**) across six subfamilies of angiosperms using DNA sequences from 31 conserved mitochondrial PCGs (**Figure 6**). The shared PCGs include *atp1*, *atp4*, *atp6*, *atp8*, *atp9*, *ccmB*, *ccmC*, *ccmFC*, *ccmFN*, *cob*, *cox1*, *cox2*, *cox3*, *matR*, *mttB*, *nad1*, *nad2*, *nad3*, *nad4*, *nad4L*, *nad5*, *nad6*, *nad7*, *nad9*, *rpl16*, *rps1*, *rps3*, *rps4*, *rps7*, *rps12*, and *rps13*. The mitochondrial genomes of two species from the *Cyperoideae* subfamily were used as outgroups. The phylogenetic topology inferred from mitochondrial DNA aligns with the most recent classification system proposed by the Angiosperm Phylogeny Group (APG). The *S. viridis* ecotype Fangshan green foxtail millet is categorized under the *Poales* order, *Poaceae* family, and *Panicoideae* subfamily, as resolved by this phylogenetic framework.

**Figure 7:**
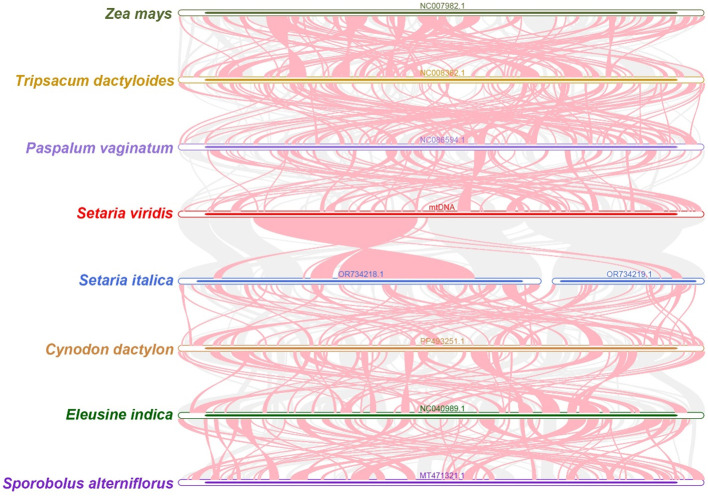
Synteny analysis of the *S. viridis* mitochondrial genome. The bars represent the mitogenomes, while the ribbons illustrate the homologous sequences between neighboring species. Red areas highlight the locations of reversals, while gray areas indicate regions of strong homology. Blocks shared by species that are less than 0.5 kb in length are excluded, and regions lacking a common block are unique to specific species.

Within this subfamily, a notable number of homologous collinear blocks were identified across species (**Supplementary Table 8**). As depicted in **Figure 7**, the regions marked by red arcs represent inverted segments, while the gray regions indicate areas with favorable homology. To optimize the result presentation, syntenic blocks shorter than 0.5 kb were excluded from the final visualization. The results revealed that the arrangement order of mitochondrial genome syntenic blocks among these 8 species is inconsistent. The mitochondrial genome of Fangshan green foxtail millet has undergone extensive genomic rearrangements relative to its closely related species. This indicates a dynamic evolutionary process that has shaped the mitochondrial genomic architecture in these lineages. Such rearrangements may potentially impact the regulation of mitochondrial gene expression and functional coordination, providing insights into the evolutionary divergence and adaptive evolution of these plant species at the mitochondrial genomic level.

### 3.7 RNA editing events

**Figure 8:**
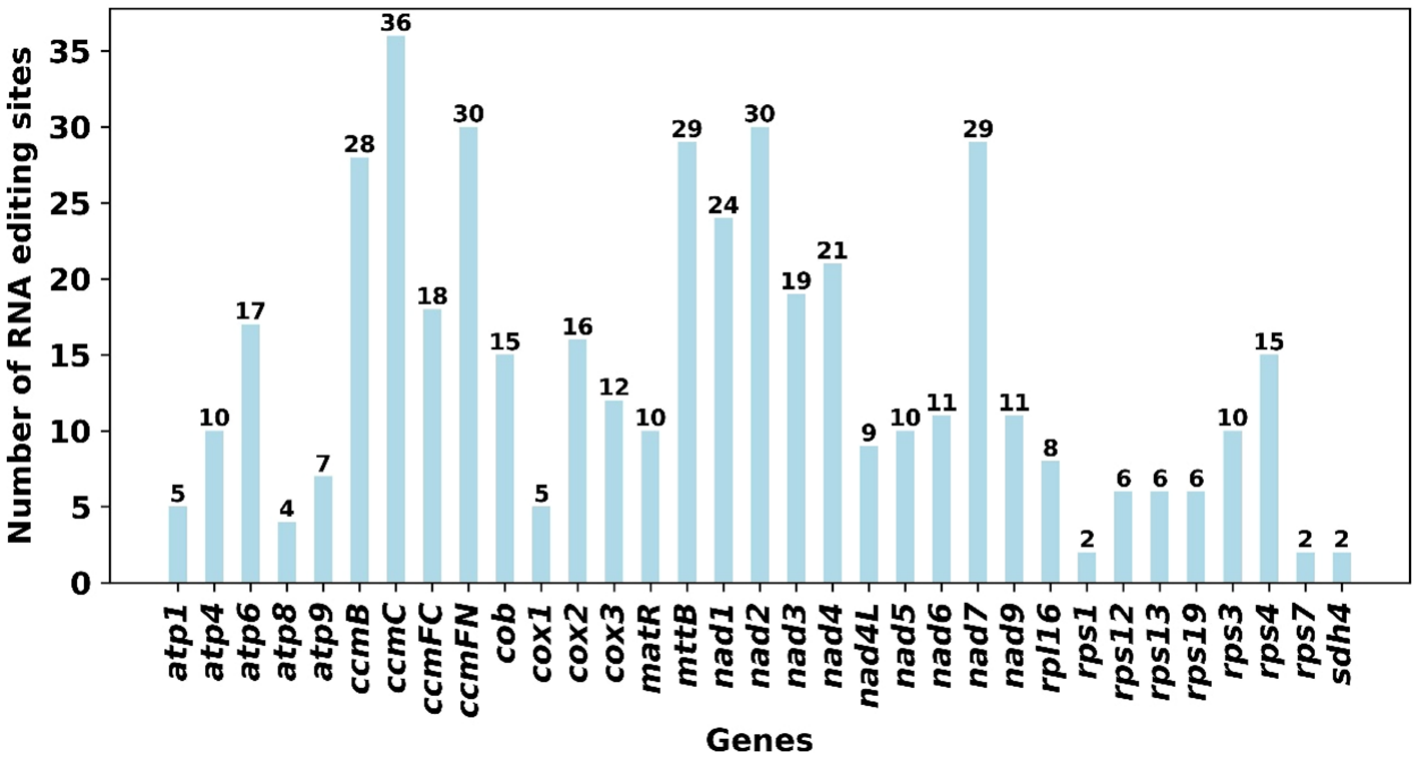
Number of RNA editing sites identified in each PCG of the *S. viridis* mitochondrial genome.

RNA editing events were interrogated across 33 unique PCGs within the mitochondrial genome of Fangshan green foxtail millet (**Supplementary Table 9**). A stringent cutoff value of 0.9 was enforced for prediction reliability. Under this criterion, a total of 463 putative RNA editing sites were identified across the 33 mitochondrial PCGs, with all events conforming to the canonical C-to-U transition pattern, consistent with the typical RNA editing modality in plant mitochondria as documented in mitochondrial genomics research (Zhang et al., 2024). Among the mitochondrial genes, the *ccmC* gene was identified as the most extensively edited locus, containing 36 RNA editing sites. The high frequency of editing within *ccmC* may potentially influence its functional output at the translational level, considering the role of RNA editing in refining mitochondrial gene expression. Further substantiating this, the *ccmFN* and *nad2* genes each exhibited 30 RNA editing sites (**Figure 8**). These differential editing patterns across genes suggest a gene-specific regulatory mechanism governing mitochondrial transcript maturation, which likely influences the proteomic composition and energy metabolism of mitochondria in *S. viridis*. This finding mirrors the functional implications of RNA editing observed in the Ventilago leiocarpa mitochondrial genome analysis, where RNA edits were shown to affect mitochondrial gene expression and protein functionality.

## 4 Discussion

Mitochondria are indispensable for eukaryotic life, generating ATP through oxidative phosphorylation and supplying critical metabolic intermediates (Chen et al., 2017; Gray, 2012; Bi et al., 2022; Liu et al., 2024). Plant mitochondrial genomes exhibit exceptional structural plasticity, undergoing frequent recombination, inversions, and incorporation of foreign sequences, leading to rapid structural evolution and considerable variability in genome size and arrangement (Gupta and Brian Golding, 1996; Choi et al., 2019; Shen et al., 2022; Wynn and Christensen, 2019). This genomic fluidity contrasts sharply with the compact and stable mtDNA observed in animals. Understanding the structural organization and evolutionary dynamics of plant mtDNA is therefore crucial for elucidating energy metabolism, growth, development, and stress responses in plants (Li et al., 2011). However, the specific mechanisms and functional consequences of this plasticity, particularly within the Poaceae family, remain poorly characterized. In this study, the ‘Fangshan’ ecotype of the *S. viridis* was selected as the research subject as a targeted effort to address this gap. This ecotype was originally collected from Fangshan District, Beijing, China (39°35’N, 116°05’E), where it grows in a temperate semi-arid hilly region. Phenotypically, it exhibits dwarf stature (30-50 cm in height), profuse tillering, and remarkable tolerance to both drought and nutrient deficiency, rendering it an ideal model material for C4 photosynthesis research.

Plant mitochondrial genomes exhibit extraordinary structural dynamism, deviating sharply from the conserved architecture observed in animal systems (Moller et al., 2021; Schuster and Brennicke, 1994; Xia et al., 2023). In the size, mitogenomes exhibit unparalleled diversity across angiosperms, ranging from the compact 66 kb genome in *Viscum scurruloideum* to the colossal 11.3 Mb configuration in *Silene conica*, largely driven by repetitive element proliferation and foreign DNA integration (Skippington et al., 2015; Sloan et al., 2012). While frequently depicted as single circular molecules, empirical evidence reveals extensive conformation diversity, including fragmented multi-chromosomal structures (*Fagopyrum esculentum*: 10 chromosomes; *Aegopodium tibiscum*: 6 chromosomes), linear isoforms (*Lactuca sativa*), and branched networks (*Quercus acutissima*) (Kozik et al., 2019; Lai et al., 2022; Liu et al., 2022a; Logacheva et al., 2020; Yang et al., 2023). This plasticity arises principally from repeat-mediated recombination (large repetitive elements (>500 bp) drive genomic rearrangements), subgenomic circle formation and foreign DNA integration enabling radical size expansion (e.g., *Silene conica*: 11.3 Mb with 45.8% repeats) without augmenting gene content (Ai et al., 2023; Sloan et al., 2012). Contrastly, the *S. viridis* mitogenome assembles as a singular circular molecule of 437, 906 bp (GC: 43.96%), notably more compact than its *Panicoideae* relatives *Pennisetum glaucum* (474 kb) and *P. miliaceum* (515 kb). This streamlined architecture arises from controlled expansion of repetitive sequences (28.7% of genome), contrasting with the long terminal repeat (LTR)-dominated inflation observed in *Silene conica* (45.8% repeats) and *Poa pratensis* (42.5%) (Ai et al., 2023; Sloan et al., 2012).

In contrast, the *S. viridis* mitogenome assembles as a single circular molecule of 437, 906 bp, notably more compact than its Panicoideae relatives *Pennisetum glaucum* (474 kb) and *Panicum miliaceum* (515 kb). This streamlined architecture can be attributed to two main factors. First, repeat content: dispersed repeats (493 pairs) and tandem repeats (33 loci) together account for approximately 28.7% of the genome, substantially lower than the repeat in *Silene conica* (45.8%) and *Poa pratensis* (42.5%) (Ai et al., 2023; Sloan et al., 2012). Second, foreign DNA integration: MTPT fragments total 13, 972 bp, representing only 3.19% of the mitogenome. Thus, the restrained proliferation of both repetitive sequences and horizontally transferred DNA contributes to the compact architecture of the *S. viridis* mitogenome. This restraint suggests that the *S. viridis* mitogenome has evolved under selective pressures favoring genomic economy while retaining sufficient repetitive content to support essential homologous recombination. The moderate repeat content (28.7%) may represent an “optimal balance” for Panicoideae species: enough to drive necessary conformational dynamics for replication and gene expression, yet not so excessive as to incur the metabolic cost of maintaining large amounts of non-coding DNA.

From a functional perspective, the compact yet recombination-competent architecture of the *S. viridis* mitogenome may be particularly advantageous for C4 grasses, which demand rapid and flexible energy metabolism in response to fluctuating light, temperature, and water availability. The ability to generate alternative conformations through repeat-mediated recombination could allow rapid adjustments in mitochondrial gene expression without altering gene content, a potential mechanism for optimizing respiratory efficiency under stress conditions. Furthermore, the observed GC content (43.96%) falls within the typical range for grass mitogenomes and may influence both replication fidelity and RNA editing efficiency, as higher GC content has been associated with increased C-to-U editing site recognition in plant mitochondria. Notably, comparative analysis within the Panicoideae subfamily reveals that mitogenome size variation is not strictly correlated with phylogenetic relatedness. For example, despite their close relationship, *S. viridis* (438 kb) and *S. italica* (reported range 450-470 kb) differ by approximately 10-15% in genome size, suggesting that rapid size fluctuations can occur even between congeneric species. This observation aligns with the emerging view that plant mitogenome size is driven more by stochastic repeat proliferation and deletion events than by adaptive selection alone. Importantly, the relatively compact size of the *S. viridis* mitogenome, combined with its complete set of 33 protein-coding genes and full complement of tRNAs, demonstrates that genome streamlining does not compromise functional completeness.

Gene annotation reveals 33 unique protein-coding genes in the *S. viridis* mitogenome, including 24 core OXPHOS components: 5 ATP synthase subunits, 9 NAD dehydrogenases, 3 cytochrome cox oxidases, and cob/ccm/matR genes, alongside 20 tRNAs and 3 rRNAs. This result demonstrates conserved respiratory functionality despite structural divergence. Critically, the identification of conserved loci (e.g., *cox1*-*atp9* hotspot) shared with *S. italica* validates Poaceae-specific mechanisms for maintaining genomic integrity during structural reorganization (Zhang et al., 2024). Meanwhile, intracellular gene transfer emerges as a key modulator of mitogenome evolution. Our analysis identifies 31 mitochondrial plastid DNA transfer (MTPT) fragments totaling 13, 972 bp (3.19% of mtDNA), including the functionally intact *ndh*J and nine tRNAs (e.g., *trnM-CAU*, *trnW-CCA*). The acquisition of plastid-derived tRNAs likely compensates for losses or deficiencies in the native mitochondrial tRNA set, a phenomenon previously documented in Clematis and Photinia (Liu et al., 2022b; Wang et al., 2023). More intriguingly, the presence of an intact *ndhJ* gene raises the possibility of neofunctionalization within the mitochondrial electron transport chain, potentially contributing to respiratory flexibility, an advantageous trait for C4 grasses adapting to variable environments. Simultaneously, 493 dispersed repeats, including an unprecedented 8, 135 bp palindromic repeat and 816 bp forward repeat, drive homologous recombination. These repeats facilitate dynamic isoform generation, evidenced by 234 palindromic and 239 forward repeat pairs, while maintaining core OXPHOS functionality through conserved gene synteny (24 respiration genes preserved with 99.1% identity to *S. divaricata*) (Ni et al., 2022). The co-occurrence of extensive SSRs (111 loci with 38.74% mono-/di-nucleotide repeats) and tandem repeats (33 loci) further contribute to the size and structural variation in mitogenomes (Qu et al., 2022). Collectively, the *S. viridis* exemplifies “functional buffering” through MTPT-driven gene recruitment and repeat-modulated genome fluidity, a dual-strategy adaptation advancing C4 grass resilience.

RNA editing further shapes the functional variant of plant mitogenomes, serving as a critical post-transcriptional mechanism that compensates for genomic mutations and optimizes protein folding under environmental stress. In the *S. viridis*, we identified 463 C-to-U editing sites across all 33 protein-coding genes, a frequency (14.0 sites/gene) exceeding model eudicots (*Arabidopsis*: 441 sites total; ~10.5 sites/gene) yet remaining below basal angiosperms (*Gossypium*: 488 sites) (Bi et al., 2016; Unseld et al., 1997). The intermediate editing frequency observed in the *S. viridis* may reflect an evolutionary position between relatively low-editing eudicots and high-editing basal angiosperms, although lineage-specific regulatory divergence cannot be ruled out. Strikingly, the *ccmC* gene exhibits the highest editing density among all PCGs, with 36 identified sites. This pattern diverges from the typical predominance of *nad* or *ccmFN* genes observed in many angiosperms, as documented in *Angelica biserrate* and *Momordica charantia* (Niu et al., 2023; Wang et al., 2024).

Given that the *S. viridis* is a model C4 plant, its photosynthetic carbon concentrating mechanism imposes a high ATP demand on mitochondria, particularly in bundle sheath cells where NAD-malic enzyme-type decarboxylation occurs. The *ccmC* gene encodes a critical component of the cytochrome c maturation pathway, which is essential for the assembly of respiratory complex III. The unusually high editing density in *ccmC* may therefore reflect a functional requirement to fine-tune cytochrome c biogenesis, thereby optimizing electron transport chain efficiency to meet the elevated energy demands of C4 photosynthesis. We speculate that extensive C-to-U editing in *ccmC* could enhance heme-binding affinity or stabilize transmembrane domains, enhancing complex III activity and ATP production under high-energy flux. This hypothesis requires experimental validation, such as comparative analysis of edited versus unedited *ccmC* transcripts in C3 versus C4 systems.

Furthermore, *ccmFN* and *nad2* each contain 30 editing sites. The high editing frequency in *ccmFN*, supports a coordinated regulatory mechanism for the entire cytochrome c biogenesis pathway. The extensive editing in *nad2*, a core subunit of mitochondrial complex I, suggests that RNA editing may also optimize NADH dehydrogenase function to sustain efficient electron flow into the respiratory chain. The differential editing patterns across genes suggest a gene-specific regulatory mechanism governing mitochondrial transcript maturation, which likely influences the proteomic composition and energy metabolism of mitochondria in *S. viridis*.

From a broader perspective, disruption or reduction of *ccmC* RNA editing could have profound consequences for mitochondrial respiratory capacity. Loss of specific editing sites in *ccmC* has been associated with impaired cytochrome c synthesis, reduced assembly of complex III, and increased reactive oxygen species accumulation in other plant systems. By analogy, mutations affecting the 36 editing sites in the *S. viridis ccmC* might lead to compromised energy metabolism, reduced growth vigor, or altered stress sensitivity. Such changes could be particularly detrimental in C4 grasses, which require highly efficient electron transport chains to support the ATP demands of the C4 carbon-concentrating mechanism. Conversely, natural variation in editing efficiency at specific *ccmC* sites could serve as a source of quantitative phenotypic diversity, potentially influencing adaptation to different environmental niches.

Nevertheless, the unusually high editing density observed in *ccmC* is consistent with a broader emerging view: RNA editing in plant mitochondria serves not merely as a mutation repair mechanism, but as an adaptive regulatory layer that fine-tunes gene expression to meet the physiological demands of specific metabolic pathways, including the high energy requirements of C4 photosynthesis. Future experimental validation, or transgenic complementation assays with edited versus unedited *ccmC*- will be essential to elucidate the causal relationships between specific C-to-U conversions in *ccmC* and mitochondrial function in *S. viridis*.

## 5 Conclusion

This study presents the first complete assembly of the mitochondrial genome of the *S. viridis*. The genome is a single circular molecule with a total length of 437, 906 bp and a GC content of 43.96%, encoding 33 protein-coding genes, 20 tRNAs, and 3 rRNAs. RNA editing prediction identified 463 C-to-U editing sites, with the highest frequencies found in *ccmC* (36 sites), *ccmFN* and *nad2* (30 sites each), suggesting their potential involvement in post-transcriptional regulation of C4 photosynthesis. This study provides a critical mitochondrial genomic resource for the evolution and molecular breeding of *Setaria* and the *Panicoideae* subfamily.

## Data Availability

The datasets generated for the purposes of this research have been duly deposited into GenBank, and are accessible via the accession numbers PZ381393 for mitochondrial genome and PZ381390 for chloroplast genome. Furthermore, the raw data obtained from both Oxford Nanopore Technologies (ONT) and Illumina platforms have been submitted to the National Center for Biotechnology Information (NCBI), and can be accessed using the respective accession numbers: PRJNA1462943, SAMN59130380, and SRR38423282 (Illumina short reads), and SRR38423283 (Oxford Nanopore long reads).

## Supplementary material

Supplementary Table 1 Protein-coding genes in the mitochondrial genome of the *S. viridis*.

Supplementary Table 2 SSRs in the mitochondrial genome of the *S. viridis.*

Supplementary Table 3 Tandem repeat sequences in the mitochondrial genome of the *S. viridis.*

Supplementary Table 4 Dispersed repeat sequences in the mitochondrial genome of the *S. viridis.*

Supplementary Table 5 Length and sequencing depth of each node.

Supplementary Table 6 The homologous DNA fragment in the *S. viridis’*s mitochondrial genome.

Supplementary Table 7 List of species and their corresponding GenBank accession utilized for phylogenetic assessment.

Supplementary Table 8 Synteny analysis of the *S. viridis.*

Supplementary Table 9 RNA editing events detected in the mitochondrial protein-coding genes of the *S. viridis.*
